# Single-nucleus transcriptomic analysis identifies a putative Purkinje fiber-associated remodeling program after myocardial infarction

**DOI:** 10.1186/s12872-026-06409-0

**Published:** 2026-08-13

**Authors:** Peiran Song, Zemin Kang, Haiyan Hou, Ziye Zhang, Kai Tang, Dongdong Zhao, Yuan Zhong, Hailing Li

**Affiliations:** 1 Department of Cardiology, Shanghai Tenth People’s Hospital, Tongji University School of Medicine, Shanghai, China; 2 Tongji University School of Medicine, Shanghai, China; 3 Department of Cardiology, Shanghai First People’s Hospital, Shanghai Jiao Tong University School of Medicine, Shanghai, China

**Keywords:** Purkinje fiber, Transitional PFs, Myocardial infarction, Secondary analysis, Single-nucleus RNA sequencing, Metabolic remodeling, Fibroblast, CellChat

## Abstract

**Background:**

Purkinje fiber (PF) remodeling may contribute to post-infarction ventricular arrhythmia, but rare conduction-associated cardiomyocyte states in injured human myocardium remain incompletely characterized. We use TsPF to denote transitional PFs, a study-specific transitional Purkinje fiber subpopulation resolved by cardiomyocyte re-clustering and characterized by an intermediate transcriptional phenotype. In this study, transitional refers to the observed expression phenotype; temporal progression, lineage conversion, and anatomical Purkinje localization require separate evidence.

**Methods:**

We performed a secondary analysis of the human myocardial infarction atlas reported by Kuppe et al. The analyzed discovery matrix was derived from 29 single-nucleus RNA-sequencing libraries representing 28 tissue specimens from 20 individuals (4 donor controls and 16 individuals with myocardial infarction). After quality control and Seurat/Harmony integration, ventricular cardiomyocytes were re-clustered. PF/TsPF annotation, MiloR, high-dimensional weighted gene co-expression network analysis (hdWGCNA), scMetabolism, PF-anchored pseudotime, and CellChat were evaluated with an explicit separation between abundance testing, descriptive molecular patterns, and model-derived hypotheses.

**Results:**

The quality-controlled matrix contained 172,522 nuclei and 28,122 genes. All MiloR neighborhoods had a spatial false discovery rate (SpatialFDR) of at least 0.05. A secondary tissue-sample-level TsPF: mature-PF ratio was lower in myocardial infarction than control in the displayed comparison (unadjusted Wilcoxon *P* = 0.0345); several tissues came from the same donors, and mature-PF denotes the PF-labeled denominator. Using β = 5, hdWGCNA resolved nine colored modules; the selected blue module contained 1,050 genes, including NT5C, ITPA, GUK1, and ADSS1. TsPF-labeled nuclei showed higher nucleotide-metabolism scores (average log2 fold change = 0.9982; Bonferroni-type adjusted *P* = 4.63 × 10 − 27; cell-level comparison). CellChat network summaries showed 1,191 versus 805 inferred interactions and aggregate strength 0.095 versus 0.027 in control versus myocardial infarction; these values are descriptive model outputs rather than donor-level differential tests.

**Conclusions:**

This secondary analysis prioritizes a putative PF-associated transcriptional remodeling program for follow-up. The abundance result remained negative after SpatialFDR correction, while the co-expression, metabolic, ordering, and communication analyses provide candidates for hypothesis-driven work. Establishing a distinct TsPF state and its functional relevance will require independent donor-aware, spatial/histological, molecular, and electrophysiological validation.

**Supplementary Information:**

The online version contains supplementary material available at https://doi.org/10.1186/s12872-026-06409-0.

## Background

Myocardial infarction (MI) produces electrical, structural, inflammatory, and stromal remodeling that can sustain ventricular arrhythmia [1, 2]. Loss of viable myocardium and heterogeneous fibrosis slow and fragment impulse propagation, while surviving excitable tissue at infarct borders can participate in re-entry [3–5]. These mechanisms are well established at tissue scale, but the molecular responses of specialized human conduction-associated cardiomyocytes remain difficult to resolve because they are rare and anatomically restricted.

Purkinje fibers form a rapidly conducting subendocardial network and can act as triggers or components of post-infarction ventricular tachycardia circuits [6, 7]. Experimental and pathological studies show that subendocardial Purkinje cells can survive acute ischemic injury while acquiring electrophysiological and ultrastructural abnormalities, and that infarction can alter the spatial organization of the Purkinje network [8, 9]. Recent work also links preservation or regeneration of the conduction system to arrhythmia susceptibility after MI [10]. These observations motivate molecular study of PF-associated remodeling, while the human post-infarction transcriptional state remains unresolved.

Single-cell studies have established developmental conduction-system transcriptional programs [11–13], and human cardiac single-cell and spatial atlases provide broader anatomical context [2, 14, 15]. Recent transcriptomic and machine-learning studies have also nominated diverse MI- or cardiomyopathy-associated genes [16–25]. Adult human PF responses after infarction nevertheless remain unresolved. The spatial multi-omic atlas of Kuppe et al. offers an opportunity for targeted secondary analysis, although PF biology was outside the principal scope of that single source resource [2].

We therefore asked whether ventricular-cardiomyocyte re-clustering could identify a PF-associated transcriptional compartment and whether abundance, co-expression, metabolic, ordering, and ligand–receptor analyses could generate a coherent and testable remodeling hypothesis. We use TsPF to denote transitional PFs, the cells comprising one study-specific, unsupervised transitional Purkinje fiber subpopulation adjacent to the PF cluster in the analyzed transcriptomic manifold. The label is operational and limited to the observed transcriptional phenotype; temporal direction, lineage, anatomical identity, biomarker performance, and therapeutic relevance require independent evidence.

## Methods

### Study design and data source

This study was a secondary bioinformatics analysis of the human MI single-nucleus and spatial multi-omic resource reported by Kuppe et al. [2]. The complete source project comprised 31 tissue specimens from 23 individuals; three individuals (P21–P23) were used only for validation in the source study. The principal discovery single-nucleus RNA sequencing (snRNA-seq) atlas comprised 29 libraries representing 28 tissue specimens from 20 individuals (P1–P20), including 4 non-transplanted donor controls and 16 individuals with MI. Controlled-access sequence data are available through the European Genome-phenome Archive, and processed representations are available through the Human Cell Atlas, Zenodo, and CZ CELLxGENE resources [26–32]. Repositories were rechecked on 20 July 2026.

### Sample structure and statistical independence

Individuals, tissue specimens, sequencing libraries, nuclei, metacells, and model-derived interactions were treated as distinct reporting units (Table 1). Several individuals contributed more than one tissue region or library, so analyses were interpreted with this nesting in mind. The control group comprised four individuals. The exported result files available for this revision lacked exact PF/TsPF counts for each downstream pipeline and condition-by-cell-type counts after CellChat downsampling. We left these quantities unreported rather than estimating them from plotted points. The downstream analyses are therefore exploratory and require donor-level replication before generalization.

**Table 1:** Source cohort, analyzed matrix, reporting units, and retained-output limitations

Dataset or analysis	Known source scope or rule	Reporting unit	Limitation relevant to interpretation
Complete Kuppe resource	31 tissue specimens; 23 individuals	Individual and specimen	Includes P21–P23, used only for source-study validation
Core discovery snRNA-seq atlas	29 libraries; 28 specimens; 20 individuals (4 control, 16 MI)	Individual, specimen, library	Several individuals contributed multiple tissues or libraries
Present post-QC matrix	172,522 nuclei; 28,122 genes	Nucleus for computation	Nested within specimens and individuals
MiloR	SpatialFDR; tissue-sample counts	Neighborhood and tissue sample	Exact retained PF/TsPF counts and minimum SpatialFDR unavailable
hdWGCNA	β = 5; ≤100 nuclei/metacell	Metacell and module	Exact donor/metacell n, r, kME, and adjusted P unavailable
scMetabolism	78 pathways	Nucleus-level pathway score	Three exported groups; TsPF state and MI status are confounded
PF-anchored pseudotime	Root selected by PF enrichment	Nucleus-level display coordinate	Alternative-root sensitivity could not be rerun
CellChat	Minimum 10; maximum 3,000 nuclei per cell type/condition	Model-derived interaction	Exact post-sampling counts and donor-level differential tests unavailable

### Single-nucleus preprocessing, integration, and annotation

Single-nucleus preprocessing used a Seurat-based workflow [33]. Quality metrics included nFeature_RNA, nCount_RNA, and percent.mt. Nuclei with 200–4,000 detected features and percent.mt < 5% were retained. Data were log-normalized with a scale factor of 10,000; the top 3,000 highly variable genes were selected; and the integrated object was scaled and reduced by principal-component analysis. Harmony corrected sample-level batch effects before graph construction [33, 34]. The first 30 Harmony dimensions were used, global clustering resolution was 0.3, and uniform manifold approximation and projection (UMAP) used n.neighbors = 50. The archived QC export retained informative nFeature_RNA and nCount_RNA distributions, whereas its mitochondrial-percentage panel was unusable. Revised Supplementary Figure S1 (Additional file 1) omits that panel while retaining the applied percent.mt threshold in the Methods. The resulting matrix contained 172,522 nuclei and 28,122 genes.

### Ventricular cardiomyocyte re-clustering

Ventricular cardiomyocytes were subset and re-analyzed using normalization, variable-feature selection, scaling, principal-component analysis, Harmony integration, graph clustering, and UMAP. The top 2,000 highly variable genes and the first 30 Harmony dimensions were used for cardiomyocyte-only graph construction. Clustering resolution 1.0 and UMAP n.neighbors = 30 generated the partition and visualization used for subsequent PF/TsPF analyses. Membership followed the graph partition without applying a post hoc single-gene threshold or fixed embedding-distance rule.

### PF/TsPF cluster annotation

PF/TsPF annotation was a study-specific, cluster-level interpretation. The putative PF label was assigned by reviewing coordinated cardiomyocyte and conduction-associated expression, including GJA5, IRX3, CNTN2, SCN5A, HCN4, NKX2-5, and related features, together with the local embedding context. The displayed discriminatory features included relatively higher PRELID2 and HEYL2-AS1 in the PF-labeled cluster and ATP1A3 and PRDM16 in the TsPF-labeled cluster (Fig. 3). We designate the latter cluster as the transitional Purkinje fiber subpopulation and use TsPF to denote its constituent transitional PFs in the analyzed transcriptomic manifold. Annotation relied on coordinated cluster-level evidence rather than a single marker cutoff, classifier score, or prespecified UMAP-distance threshold. In this adult post-MI analysis, transitional denotes an intermediate expression phenotype; equivalence to the developmental TsPF_CM state described by Lázár et al. [14], temporal progression, and lineage conversion remain open questions. Alternative clustering resolutions, integration choices, continuous-state models, or merging of the two clusters could change membership and downstream summaries; Table 2 separates the supporting observations from the evidence still needed.

**Table 2:** Comparison of the PF and TsPF subpopulations

Feature	PF-labeled cluster	TsPF-labeled cluster	Interpretive boundary
Definition	Graph-clustered cardiomyocyte group interpreted in PF/conduction context	Transitional Purkinje fiber subpopulation with an adjacent, intermediate PF-associated transcriptional pattern	Anatomical and histological validation remains pending
Displayed discriminatory features	PRELID2; HEYL2-AS1	ATP1A3; PRDM16	Top features describe this partition; diagnostic performance is untested
Broader annotation evidence	Coordinated cardiomyocyte/conduction-associated expression and local embedding context	PF-adjacent embedding position plus a distinct cluster-level program	Cluster-level assignment rather than a single-gene or distance rule
Downstream observations	Relative ketone-body and fat-soluble-vitamin pathway scores	Nucleotide/RNA/selenoamino/polyamine pathway scores; blue-module gene intersection	Cell-level exploratory summaries from one source atlas
Evidence still needed	Conduction anatomy and electrophysiology	MI-specific expansion, temporal progression, lineage conversion, and causal function	Alternative annotation strategies could merge or repartition both groups

### Differential abundance analysis

MiloR was applied to the cardiomyocyte subset to evaluate local abundance differences across the cellular manifold [35]. A k-nearest-neighbor graph was built from the Harmony embedding with k = 30 and d = 30; makeNhoods used prop = 0.1, k = 30, d = 30, and refined = TRUE. Cells were counted by available tissue-sample identity, and neighborhood abundance was modeled using the MI-versus-control design. Neighborhood P values were corrected using the spatial false discovery rate (SpatialFDR); all neighborhoods had SpatialFDR ≥ 0.05. A separate tissue-sample-level TsPF: mature-PF ratio comparison used an unadjusted Wilcoxon test. This secondary comparison involved imbalanced groups and repeated donors across some tissues; mature-PF denotes the PF-labeled denominator in Supplementary Figure S4 (Additional file 4).

### hdWGCNA co-expression analysis

PF- and TsPF-labeled nuclei were analyzed using hdWGCNA [36]. Genes expressed in at least 5% of selected nuclei were retained. Metacells were constructed by subtype and sample identity, with up to 100 nuclei per metacell. A signed network used soft-threshold power β = 5; dynamic tree cutting used a minimum module size of 20, and related modules were merged at cut height 0.15. Nine non-grey modules were resolved. The archived numerical output contained module membership and gene lists but lacked the full module–trait correlation matrix, exact P values, module eigengene-based connectivity (kME) values, and multiplicity-adjusted statistics. Module–trait patterns are therefore reported descriptively.

### Metabolic pathway analysis

PF/TsPF-related nuclei were analyzed using scMetabolism with the VISION backend and REACTOME metabolic pathways [37, 38]. The archived scMetabolism export contained three groups: normal_PF, myocardial infarction_PF, and myocardial infarction_TsPF; a normal_TsPF group was absent from this export. Differential pathway testing used FindAllMarkers on the metabolism assay with only.pos = TRUE, min.pct = 0.05, and logfc.threshold = 0.1. The archived nucleus-level result table contained raw and adjusted P values for 78 pathways; adjusted values were numerically consistent with a Bonferroni-type correction across the tested family. Because TsPF labeling and MI status are inseparable in this three-group contrast, these values describe cell-level group-associated pathway scores rather than independent TsPF-state or donor-level disease effects.

The archived Path7_WGCNA_Combined_Intersection export contained four genes (NT5C, ITPA, GUK1, and ADSS1) shared between the selected blue module and a previously selected pathway gene set. The retained files lacked the full upstream Path7 gene set and its selection record; accordingly, this four-gene intersection is reported only as an exploratory candidate list and was not used for statistical testing.

### Pseudotime inference

Monocle 2 was used to arrange PF- and TsPF-labeled nuclei along a transcriptional coordinate [39]. Ordering genes were selected by differential-gene testing followed by DDRTree reduction. The root was chosen after examining PF enrichment so that the PF-enriched region appeared at the low end of the displayed coordinate. This choice sets the figure orientation; re-rooting could reverse early/late labels and apparent trend direction. We therefore use the term PF-anchored pseudotime for an ordering of transcriptional variation, while lineage, temporal progression, and PF-to-TsPF conversion require independent evidence.

### Cell-cell communication analysis

CellChat was used to compare model-derived ligand–receptor communication networks between control and MI groups, emphasizing Secreted Signaling interactions in CellChatDB.human [40]. Fibroblasts were re-clustered as activated, homeostatic, WNT-related, and contaminated groups. Condition-specific objects were downsampled to a maximum of 3,000 nuclei per cell type where necessary; communication probabilities used the triMean method with trim = 0.1, and interactions with fewer than 10 supporting nuclei were filtered (min.cells = 10). The exported outputs lacked exact post-sampling counts. We treated CellChat probabilities and internal permutation support as model summaries; donor-level normal-versus-MI testing lay outside these outputs. A retained probability of zero was interpreted as a model result rather than biological absence.

### Statistical reporting and multiple testing

MiloR controlled neighborhood-level multiplicity using SpatialFDR. scMetabolism results are reported with the adjusted P values stored in the 78-pathway output, consistent with Bonferroni-type adjustment. The tissue-sample ratio test was unadjusted and secondary. The retained hdWGCNA output lacked the statistics needed to verify correction across module–trait comparisons, and the CellChat output lacked a donor-level between-condition test. We therefore report both analyses descriptively. CellChat percent changes are calculations from model probabilities.

### Interpretive framework

We used an interpretive framework to compare complementary views of the same atlas: cluster annotation, MiloR neighborhood abundance, tissue-sample composition, hdWGCNA, pathway scoring, PF-anchored ordering, and CellChat. Because these methods share one dataset, they provide complementary analyses rather than independent replication. SpatialFDR-controlled MiloR is treated as the primary abundance result; directly recoverable module sizes, pathway effect sizes, and model probabilities as quantitative descriptive results; and pseudotime direction and ligand–receptor changes as hypotheses requiring external validation. Exact values and reporting units are summarized in Additional file 11.

### Validation scope

The source study included 28 raw Visium slides and 27 post-QC spatial specimens (91,517 spots) [2]. These data share the source cohort with the snRNA-seq resource and could provide supportive signature projection. Multicellular spots, adjacent-section mapping, non-conduction-system-specific sampling, absence of a dedicated histological PF label, repeated specimens from some donors, and ambient-RNA risk in injured tissue limit definitive localization. The retained revision package lacked the complete spatial objects required for a reproducible new projection. Spatial, independent-dataset, histological, protein, and electrophysiological validation therefore remain future priorities.

### Use of an AI-assisted language tool

During revision, OpenAI Codex was used for language editing, manuscript organization, and preparation of a text-based workflow diagram. The authors reviewed and verified all revised text, numerical values, references, and interpretations. The tool did not generate or alter source data or statistical results.

## Results

### Study workflow and analyzed cohort

**Fig. 1:**
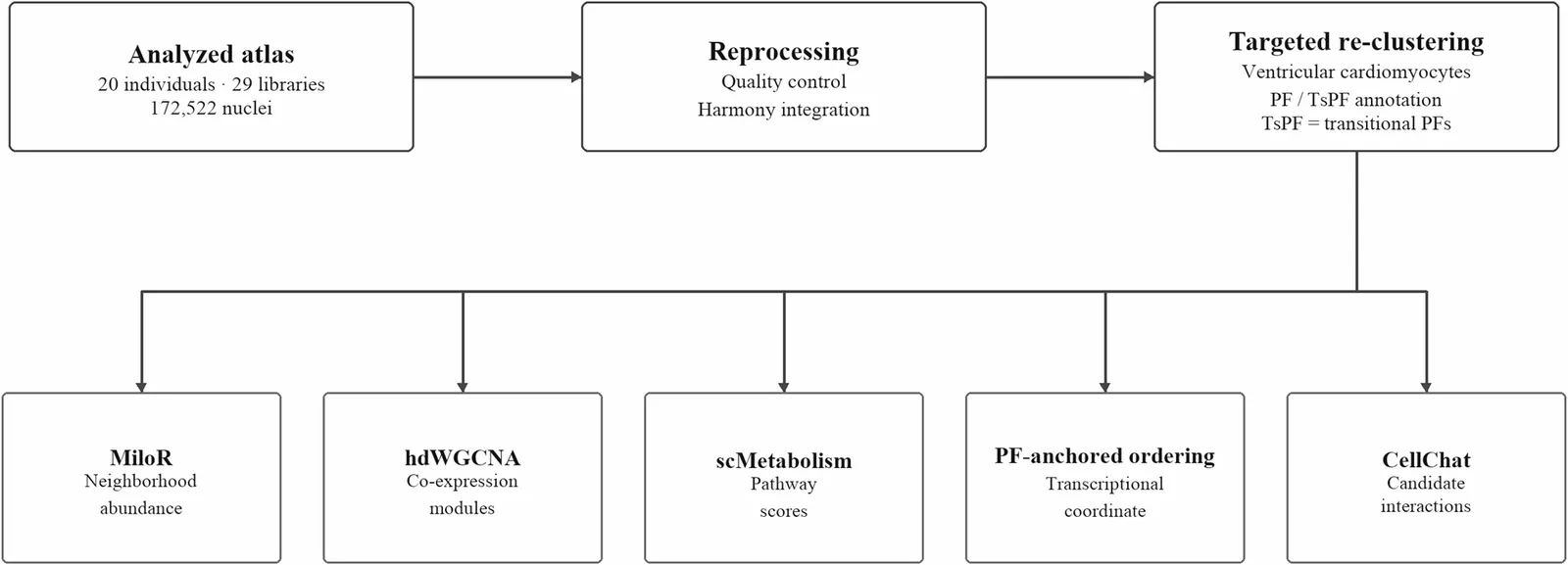
Study workflow. The analyzed core snRNA-seq atlas, cardiomyocyte re-clustering, PF/TsPF annotation, and five complementary analytical branches are shown. All branches derive from the same atlas

The targeted analysis began with the core discovery snRNA-seq atlas from 20 individuals rather than all 23 individuals in the complete Kuppe resource. The 29 libraries represented 28 tissue specimens from 4 donor controls and 16 individuals with MI. After secondary filtering and integration, 172,522 nuclei were retained. Figure 1 summarizes how PF/TsPF cluster-level annotation fed into five complementary analytical branches, all derived from this analyzed atlas.

### Global single-nucleus landscape of the cardiac dataset

**Fig. 2:**
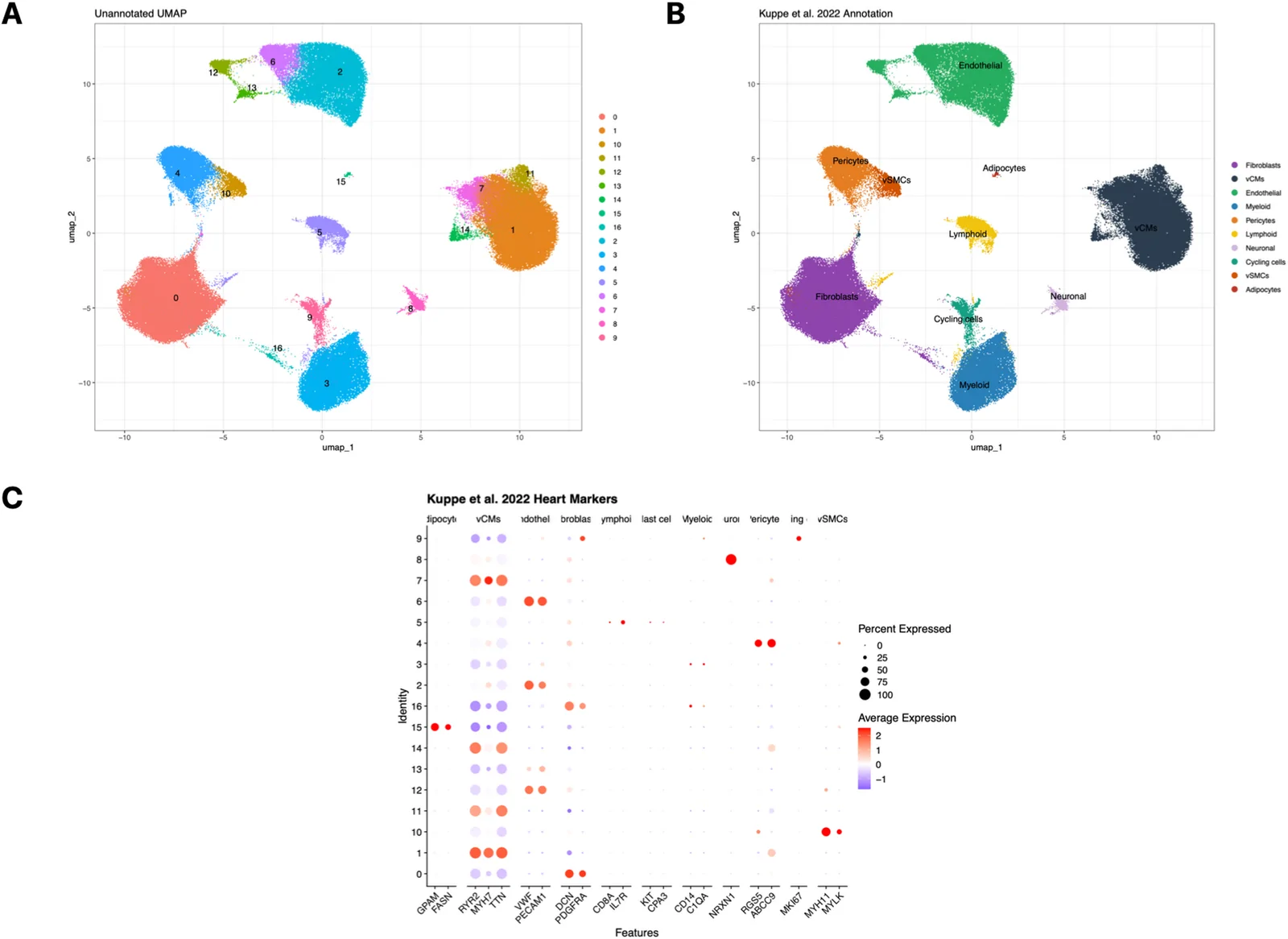
Global single-nucleus atlas. **A** Unannotated global UMAP. **B** Major cardiac cell populations after cluster-level annotation. **C** The analyzed matrix contains 172,522 nuclei from the 20-individual core discovery cohort

After quality control, the integrated atlas contained 172,522 nuclei and 28,122 genes. Major cardiac cell populations separated in UMAP space, including ventricular cardiomyocytes, fibroblasts, endothelial cells, myeloid and lymphoid cells, pericytes, smooth-muscle cells, neuronal cells, and adipocytes (Fig. 2). Revised Supplementary Figure S1 (Additional file 1) shows the retained nFeature_RNA/nCount_RNA distributions and clustering-stability tree; the unusable mitochondrial-percentage display from the archived export was omitted.

### PF and TsPF subpopulations are resolved within the cardiomyocyte compartment

**Fig. 3:**
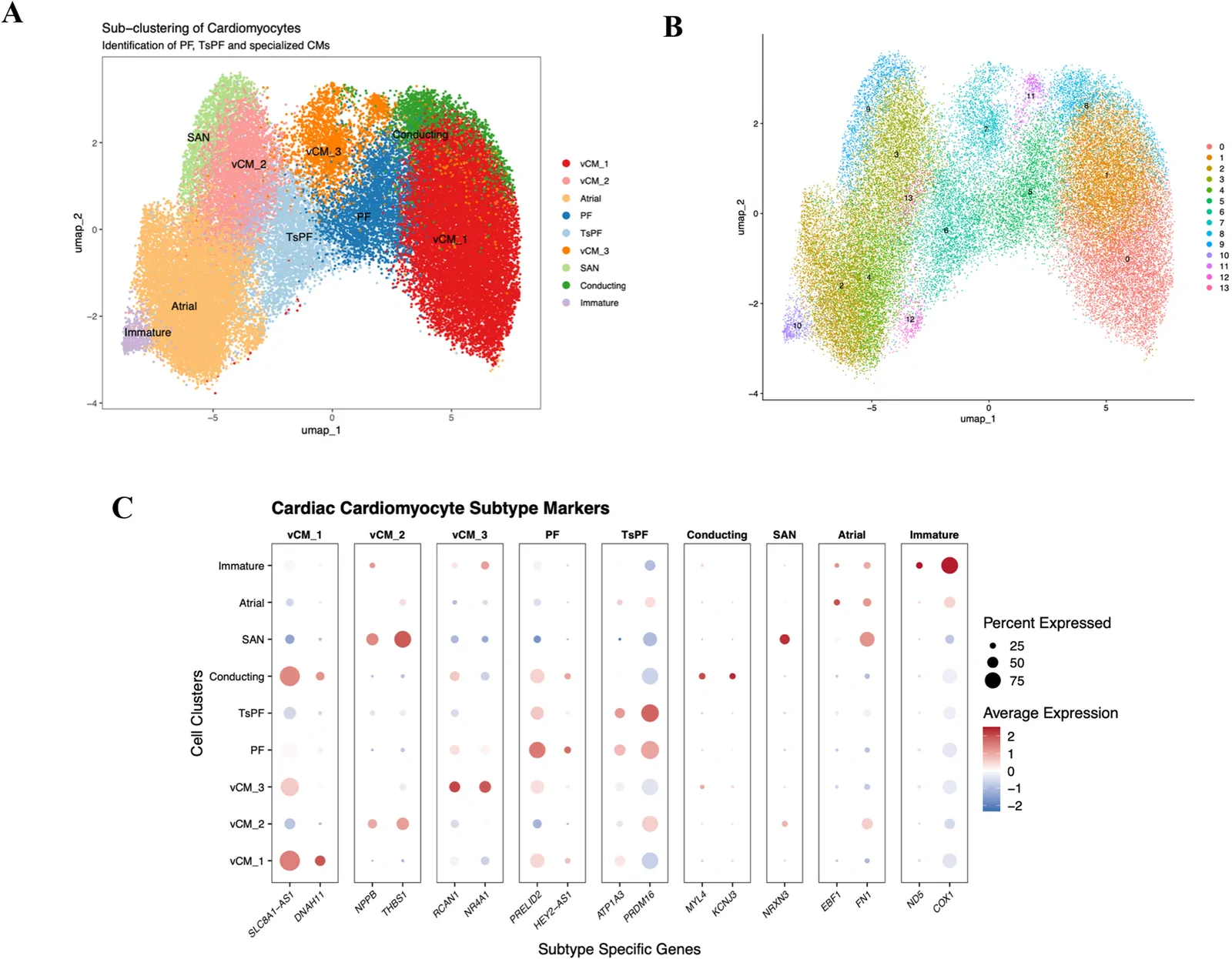
Cardiomyocyte re-clustering and PF/TsPF annotation. **A** Annotated cardiomyocyte UMAP. **B** Unsupervised graph clusters. **C** Cluster-level discriminatory-marker display. TsPF denotes the transitional PF subpopulation generated by the resolution-1.0 graph partition and interpreted from cluster-level evidence rather than a single-gene or distance cutoff

Re-clustering of ventricular cardiomyocytes resolved adjacent PF- and TsPF-labeled clusters within a broader manifold containing working ventricular, atrial-like, sinoatrial-node-like, conducting, and immature states (Fig. 3A, B). The TsPF cluster corresponds to the study-specific transitional PF subpopulation defined above. Its assignment followed the graph partition and cluster-level expression pattern rather than UMAP distance. The marker display included PRELID2 and HEYL2-AS1 for the PF-labeled cluster and ATP1A3 and PRDM16 for the TsPF-labeled cluster (Fig. 3C). These features describe the displayed partition and its intermediate transcriptional phenotype; anatomical identity, temporal progression, and lineage remain to be tested.

### Differential-abundance testing yields no evidence of TsPF expansion

MiloR showed heterogeneous local log-fold-change estimates, and all cardiomyocyte neighborhoods had SpatialFDR ≥ 0.05 (Supplementary Figure S4A, B; Additional file 4). A separate tissue-sample-level TsPF: mature-PF ratio was lower in the displayed MI group than in controls (unadjusted Wilcoxon *P* = 0.0345; Supplementary Figure S4C). Here, mature-PF denotes the PF-labeled denominator. This secondary comparison involved imbalanced groups and repeated donors across some tissues; the evidence for an MI-associated abundance difference therefore remains insufficient.

### Exploratory hdWGCNA prioritizes PF/TsPF co-expression programs

**Fig. 4:**
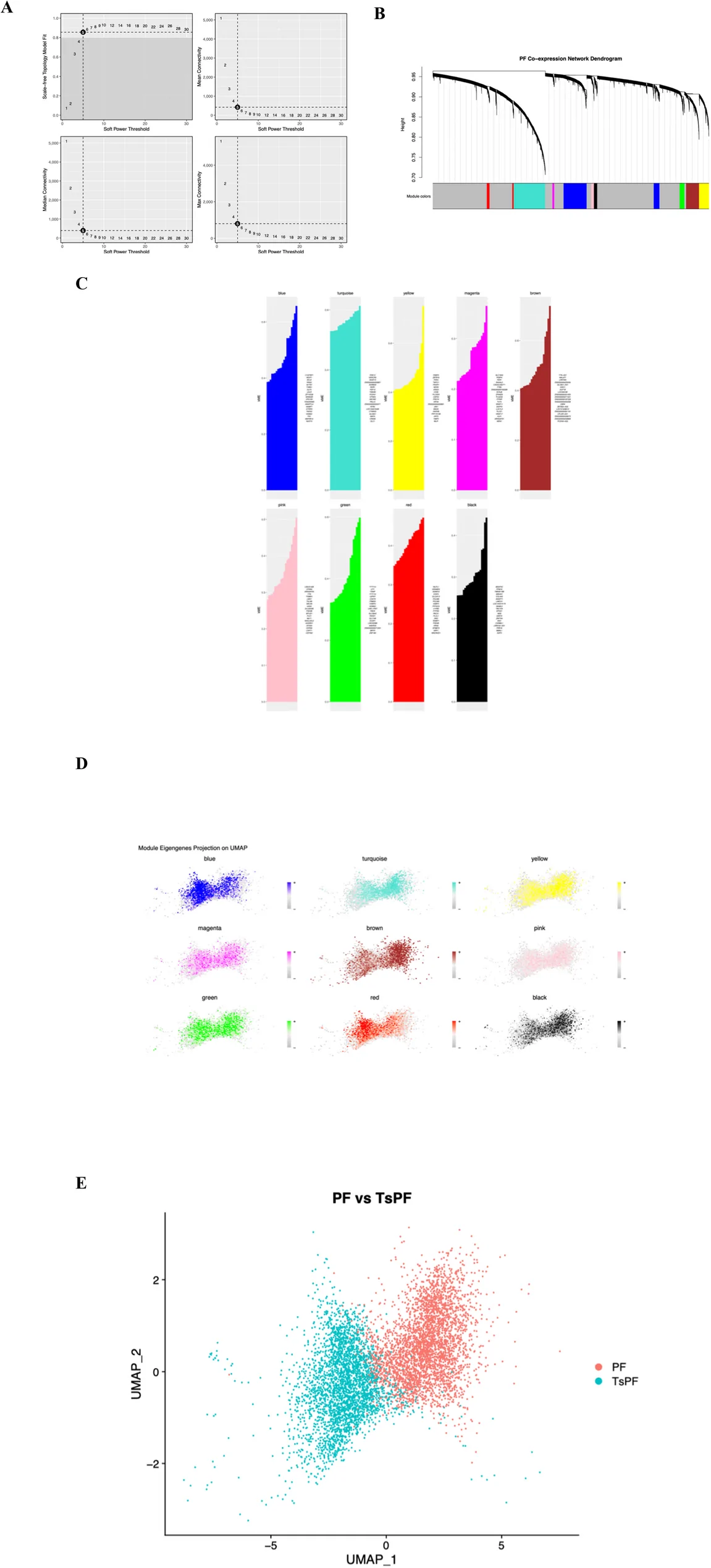
Exploratory PF/TsPF co-expression analysis. **A** Soft-threshold selection. **B** Dendrogram and module assignment. **C** Displayed module gene rankings. **D** Module-eigengene projections. **E** PF/TsPF embedding. Module–trait patterns are descriptive because exact coefficients and adjusted P values were unavailable

Using β = 5, hdWGCNA resolved nine non-grey co-expression modules across the PF/TsPF compartment (Fig. 4A–E). The selected blue module contained 1,050 unique genes. NT5C, ITPA, GUK1, and ADSS1 were present in both this module and the candidate nucleotide-related intersection (Additional files 8 and 10). The archived output lacked exact module–trait correlation coefficients, kME values, and multiplicity-adjusted P values, so the module associations are reported as exploratory patterns.

#### Metabolic remodeling is associated with PF/TsPF states

**Fig. 5:**
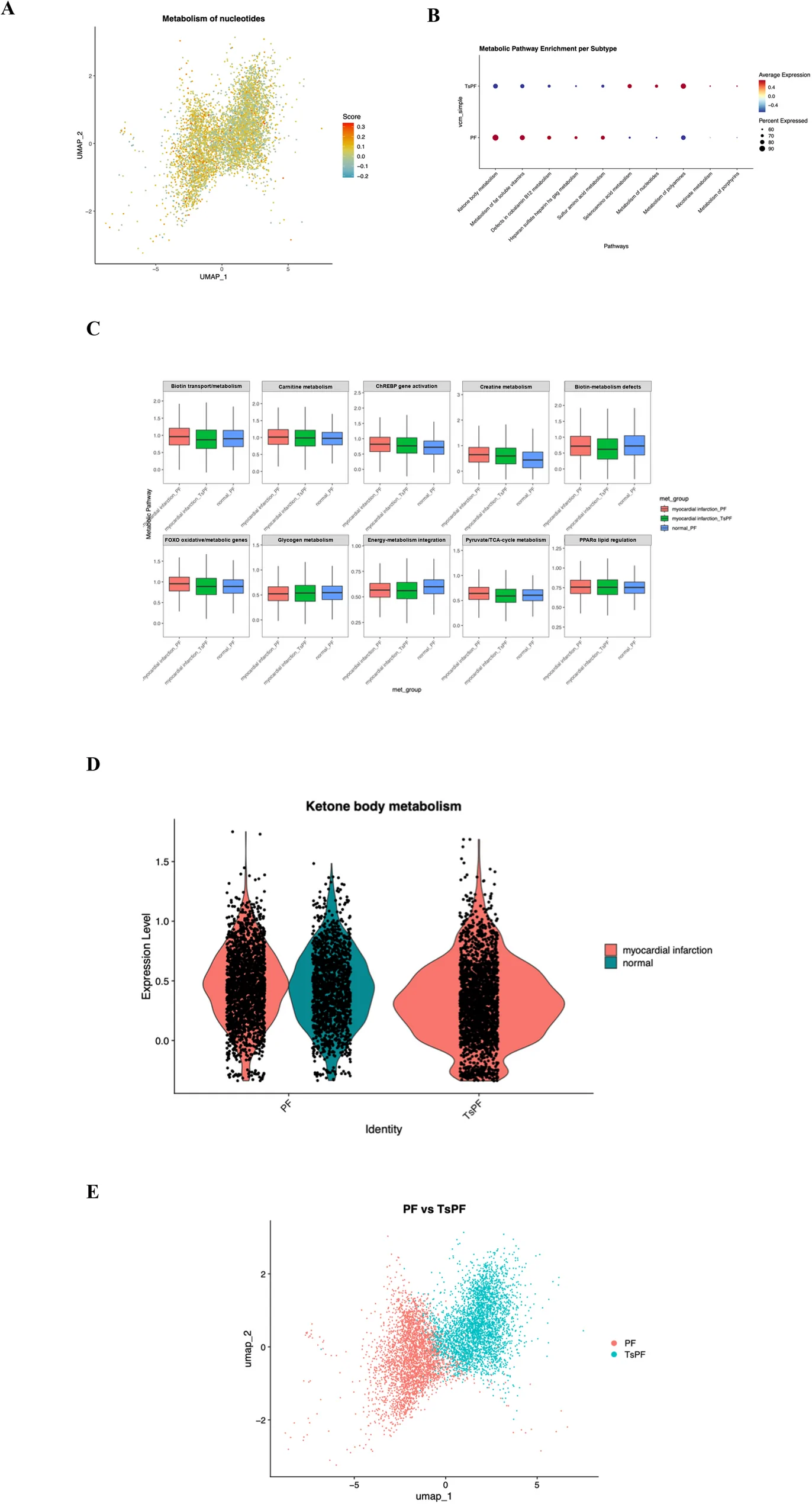
Exploratory metabolic pathway scores in PF/TsPF-labeled nuclei. **A** Representative UMAP pathway score. **B** PF/TsPF pathway-score dot plot. **C** Three-group descriptive distributions; facet headings are abbreviated for legibility. **D** Ketone-body pathway distribution. **E** PF/TsPF embedding. Adjusted P values for the 78-pathway family are reported in the text and Additional file 9. Panels summarize nucleus-level contrasts among the three exported groups

Cell-level pathway-score comparisons identified PF-associated ketone-body metabolism (average log2 fold change = 0.4661; adjusted *P* = 2.95 × 10 − 43) and fat-soluble-vitamin metabolism (0.4257; adjusted *P* = 1.37 × 10 − 18). MI_TsPF nuclei showed higher RNA metabolism (0.4008; adjusted *P* = 3.74 × 10 − 125), selenoamino-acid metabolism (1.0035; adjusted *P* = 1.49 × 10 − 49), nucleotide metabolism (0.9982; detected fractions 0.711 versus 0.596; adjusted *P* = 4.63 × 10 − 27), polyamine metabolism (0.4273; adjusted *P* = 8.34 × 10 − 24), nicotinate metabolism (1.3532; adjusted *P* = 8.50 × 10 − 9), and porphyrin metabolism (0.5927; adjusted *P* = 0.0494) (Fig. 5; Supplementary Figure S2, Additional file 2; Additional files 9 and 11). These are nucleus-level three-group contrasts in which TsPF state and MI status are confounded. Cofactor metabolism fell short of the adjusted threshold (average log2 fold change = 0.3117; adjusted *P* = 0.0536). 

### PF-anchored pseudotime displays a continuous transcriptional ordering

**Fig. 6:**
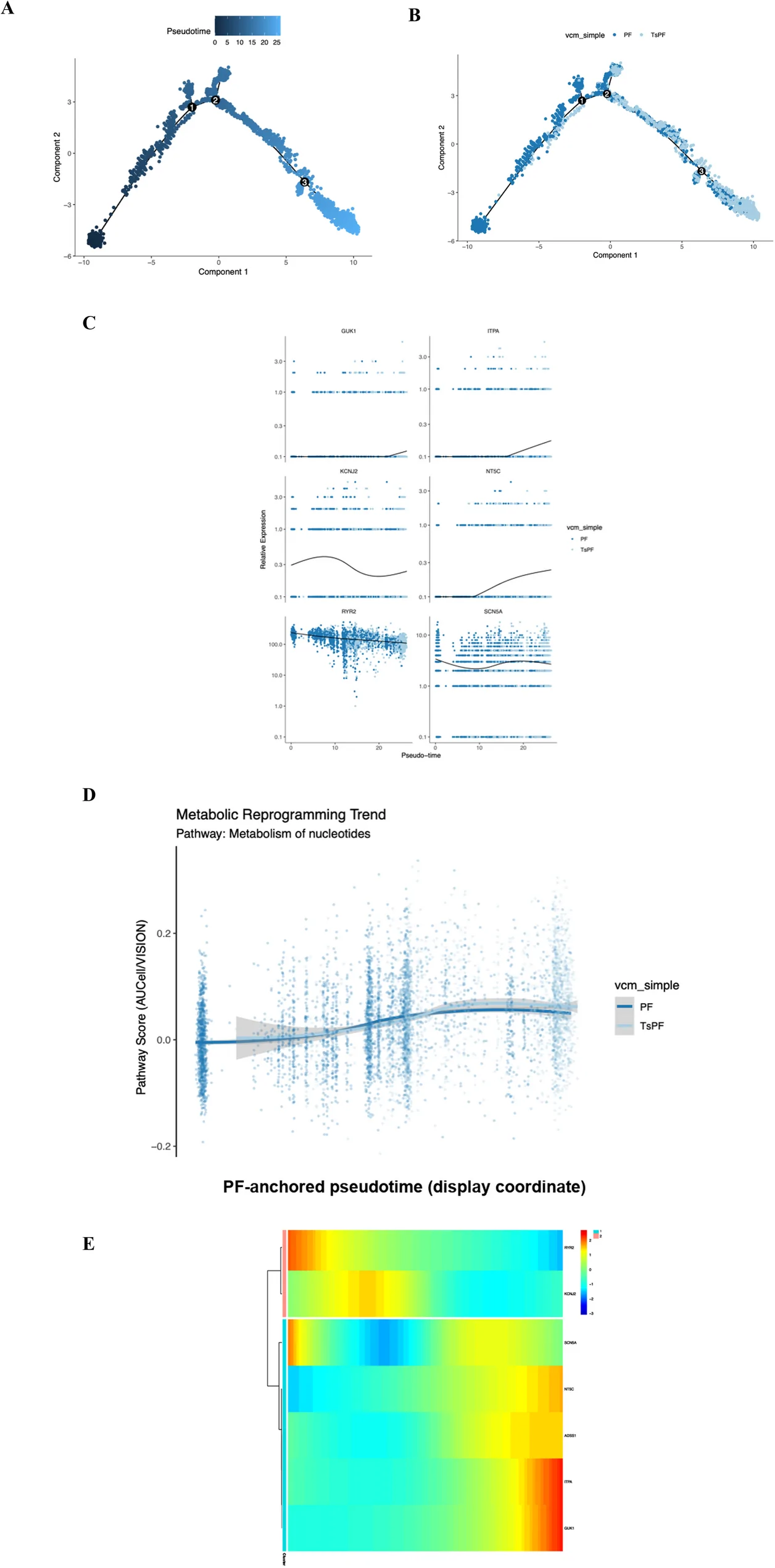
PF-anchored transcriptional ordering. **A** Monocle coordinate colored by pseudotime. **B** PF/TsPF identity. **C** Selected gene trends. **D** Nucleotide-metabolism score along the display coordinate. **E** Selected-gene heatmap. The PF-enriched root sets the visual orientation; lineage and temporal progression remain outside the inference

Monocle placed PF- and TsPF-labeled nuclei along a continuous transcriptional coordinate (Fig. 6A, B). Ion-handling and metabolic genes, including RYR2, KCNJ2, SCN5A, NT5C, ITPA, GUK1, and ADSS1, varied along the displayed ordering (Fig. 6C–E; Supplementary Figure S3, Additional file 3). Because the root was oriented by PF enrichment, the coordinate describes expression ordering; temporal direction and PF-to-TsPF conversion require independent evidence.

### Descriptive CellChat network summaries and fibroblast heterogeneity

**Fig. 7:**
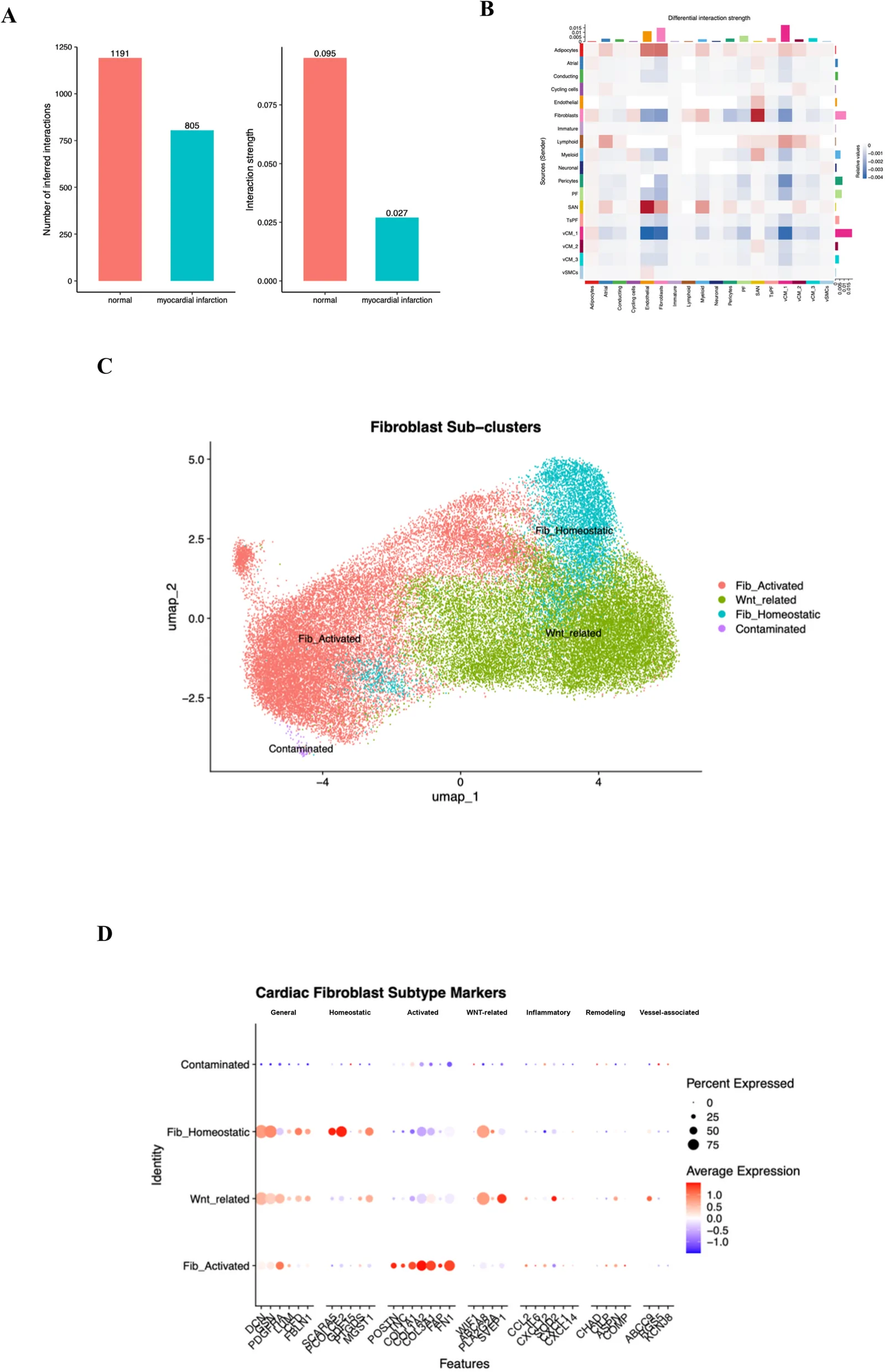
Descriptive CellChat network summaries and fibroblast heterogeneity. **A** Inferred interaction number and aggregate strength in control and MI. **B** Model-derived differential heatmap. **C** Fibroblast subclusters. **D** Fibroblast marker display. Panels A and B are model summaries rather than donor-level differential tests

At the global model-summary level, the inferred network contained 1,191 interactions in control and 805 in MI (− 32.4%), while aggregate interaction strength was 0.095 and 0.027, respectively (− 71.6%) (Fig. 7A). The exported summaries lacked donor-level P values and confidence intervals and may be affected by cell number and composition. Fibroblast re-clustering resolved activated, homeostatic, WNT-related, and contaminated groups (Fig. 7C, D). The differential heatmap is therefore used to prioritize patterns for testing (Fig. 7B).

### CellChat prioritizes candidate PF/TsPF-to-fibroblast axes

**Fig. 8:**
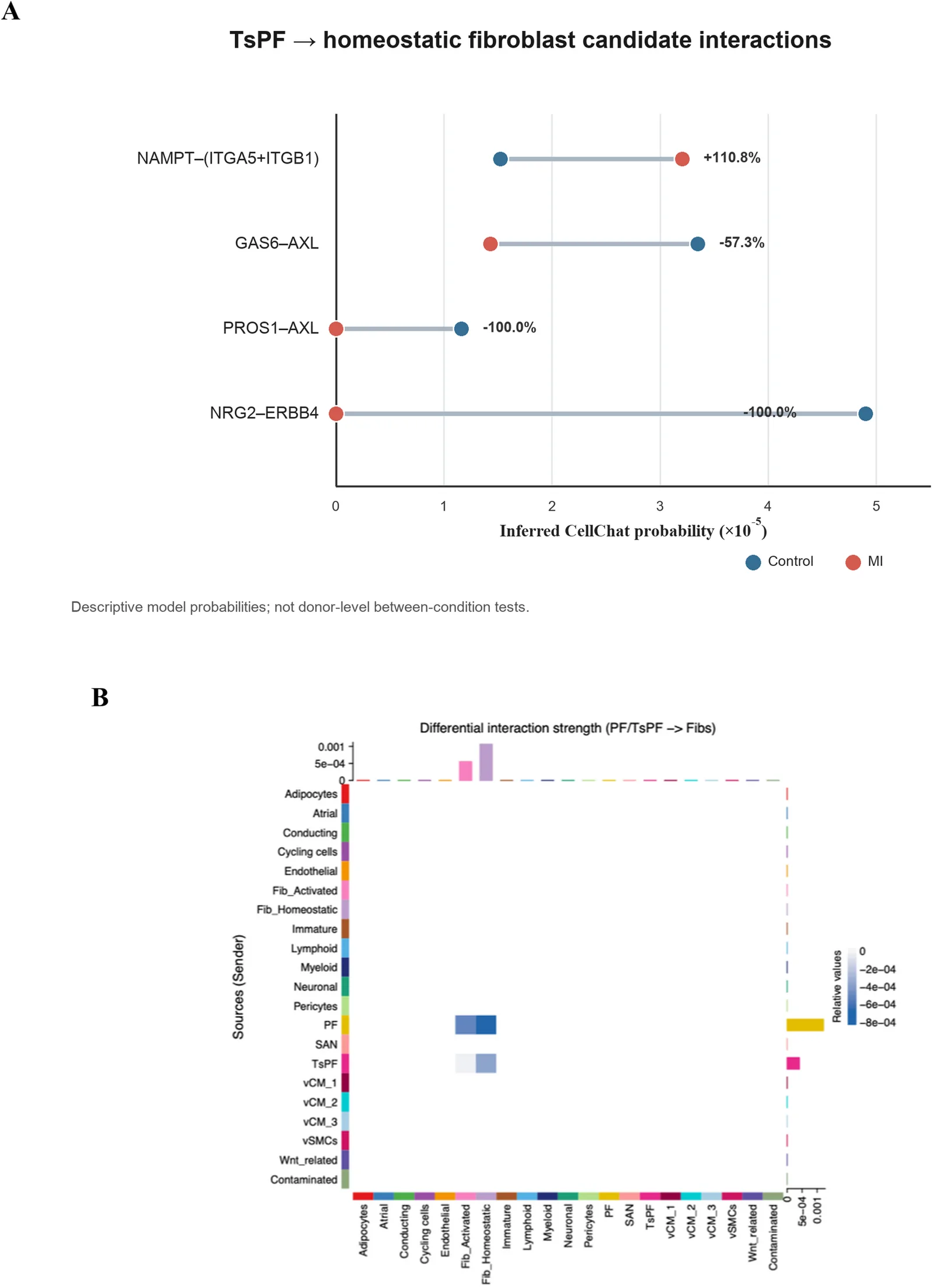
Candidate PF/TsPF-to-fibroblast communication axes. **A** Control and MI CellChat probabilities for four TsPF-to-homeostatic-fibroblast candidate interactions; percentages are descriptive changes. **B** Focused model-derived differential heatmap. Ligand–receptor activity and donor-level between-condition significance remain to be tested

In the TsPF-to-homeostatic-fibroblast comparison, the model probability for NAMPT–(ITGA5 + ITGB1) changed from 1.52128 × 10 − 5 in control to 3.20652 × 10 − 5 in MI (+ 110.8%), whereas GAS6–AXL changed from 3.34857 × 10 − 5 to 1.42977 × 10 − 5 (− 57.3%). PROS1–AXL changed from 1.16433 × 10 − 5 to 0, and NRG2–ERBB4 from 4.90001 × 10 − 5 to 0 (Fig. 8A; Additional files 5–7 and 11). A retained probability of zero is a model result rather than evidence of biological absence. The focused heatmap is presented as a prioritization view (Fig. 8B); cell-number sensitivity, donor composition, protein abundance, spatial proximity, and functional signaling remain to be tested.

## Discussion

This targeted secondary analysis identifies a PF-associated transcriptional hypothesis rather than a demonstrable expansion of a new cell population. The primary neighborhood-level abundance analysis was negative after SpatialFDR correction, and the secondary tissue-sample ratio was lower in MI but not donor-independent. The contribution is therefore a quantitative organization of co-expression, metabolic, ordering, and communication candidates within a rare conduction-associated compartment that was not the principal focus of the source atlas.

The biological context is plausible but not yet proven. PFs can survive within or near injured subendocardial tissue, acquire abnormal electrophysiology, participate in scar-related conduction and re-entry, and trigger post-infarction ventricular tachycardia [3, 5–9]. Recent experimental work further links conduction-system integrity to arrhythmia after MI [10]. These studies justify attention to PF-associated remodeling, while the present transcriptomic data contain no rhythm phenotype, conduction measurement, or direct evidence that the TsPF-labeled cluster is arrhythmogenic.

Nucleotide and related metabolic programs may be relevant to the energetic and signaling demands of electrically specialized cardiomyocytes [38, 41]. At the biochemical level, NT5C encodes a cytosolic 5′-nucleotidase; ITPA hydrolyzes noncanonical inosine triphosphates; GUK1 phosphorylates GMP to GDP; and the muscle-enriched ADSS1 enzyme catalyzes the first committed step from IMP toward AMP. Their occurrence in the 1,050-gene blue module and the candidate metabolic intersection makes them useful candidates for follow-up, although the present study provides no evidence of PF specificity or diagnostic and therapeutic utility. Donor-level replication, anatomical localization, protein or enzyme-activity measurements, association with arrhythmia phenotypes, perturbation, and electrophysiological assessment are needed. Developmental conduction-system studies provide marker context [13] but do not validate an adult post-MI TsPF state.

The PF-anchored ordering is compatible with continuous transcriptional variation between PF-like states. Because the display was oriented by PF enrichment, alternative rooting would reverse the apparent early/late trends. The coordinate therefore describes expression ordering rather than ancestry, temporal progression, or conversion, and candidate-gene trends along it should be interpreted on that basis.

The CellChat analysis prioritizes candidate axes for experimental testing. NAMPT–integrin and GAS6/PROS1–AXL patterns may relate to a remodeling microenvironment, although their model probabilities depend on cell number, donor composition, expression sparsity, and database assumptions [40]. Fibrosis can alter conduction through structural discontinuity and coupling [5, 42, 43]; determining whether PF-derived ligands affect fibroblast behavior, or fibroblasts alter PF electrophysiology, will require spatial and functional experiments.

### Limitations and validation priorities

Several limitations are intrinsic to the source dataset and cannot be resolved through reanalysis. The cohort comprised four control and 16 MI donors, some contributing multiple tissue regions, and spatial sampling was not designed for the conduction system. Visium spots are multicellular, sections are adjacent rather than cell-matched, and PF-specific histology, rhythm phenotypes, conduction measurements, and electrophysiological data were unavailable [2]. Thus, spatial projection cannot establish the anatomical or functional identity of the TsPF-labeled cluster. The metabolic export contained normal_PF, MI_PF, and MI_TsPF but no normal_TsPF group, precluding separation of TsPF-associated from MI-associated variation.

Additional constraints include unavailable pipeline-specific PF/TsPF and post-downsampling counts, no donor-aware pseudobulk or leave-one-donor-out analysis, incomplete hdWGCNA statistics, nucleus-level pathway testing, and no alternative-root sensitivity analysis. Doublets, ambient RNA, and annotation instability may also affect rare-cell results. Biological validation requires independent MI cohorts with conduction-enriched or subendocardial sampling, higher-resolution spatial mapping, histological co-localization, balanced donor-level analyses, molecular validation, and electrophysiological testing. Broader cardiac spatial atlases provide context but cannot replace matched validation [15]. This study therefore prioritizes testable hypotheses; a distinct cell type, mechanism, biomarker, or treatment target remains unproven.

## Conclusions

In summary, this secondary analysis prioritizes a putative PF-associated transcriptional remodeling program in infarcted human myocardium. The SpatialFDR-controlled abundance analysis was negative, whereas co-expression, metabolic, PF-anchored ordering, and CellChat outputs generated testable candidates. Independent donor-aware, spatial/histological, molecular, and electrophysiological studies are now needed to determine whether the TsPF-labeled cluster has a distinct anatomical and functional role after MI.

## Supplementary Information

Supplementary Material 1.

Supplementary Material 2.

Supplementary Material 3.

Supplementary Material 4.

Supplementary Material 5.

Supplementary Material 6.

Supplementary Material 7.

Supplementary Material 8.

Supplementary Material 9.

Supplementary Material 10.

Supplementary Material 11.

## Data Availability

The original human myocardial infarction atlas is available through the European Genome-phenome Archive study EGAS00001006330 and dataset EGAD00001008952, the Human Cell Atlas project e9f36305-d857-44a3-93f0-df4e6007dc97, the processed-data Zenodo record 10.5281/zenodo.6578047, and the cellxgene collection 8191c283-0816-424b-9b61-c3e1d6258a77 . The original atlas reports source code in two GitHub repositories.

## Abbreviations

PF: Purkinje fiber
TsPF: transitional PFs (the transitional Purkinje fiber subpopulation)
MI: myocardial infarction
snRNA-seq: single-nucleus RNA sequencing
UMAP: uniform manifold approximation and projection
FDR: false discovery rate
SpatialFDR: spatial false discovery rate used by MiloR
hdWGCNA: high-dimensional weighted gene co-expression network analysis
kME: module eigengene-based connectivity
PCA: principal-component analysis
EGA: European Genome-phenome Archive
